# Serum proteomics as a strategy to identify novel biomarkers of neurologic recovery after cardiac arrest: a feasibility study

**DOI:** 10.1186/s40635-016-0084-3

**Published:** 2016-05-10

**Authors:** J. Gordon Boyd, Laura J. Smithson, Daniel Howes, John Muscedere, Michael D. Kawaja, On behalf of the Canadian Critical Care Translational Biology Group

**Affiliations:** Department of Critical Care Medicine, Queen’s University/Kingston General Hospital, Kingston, Ontario Canada; Queen’s Centre for Neuroscience Studies, Queen’s University, 2nd Floor, Botterell Hall, Kingston, Ontario Canada; Department of Biomedical and Molecular Sciences, Queen’s University, Kingston, Ontario Canada; Department of Medicine (Neurology) and Critical Care, Davies 2, Kingston General Hospital, Kingston, ON K7L 2V7 Canada

**Keywords:** Cardiac arrest, Targeted temperature management, Serum proteomics, MALDI-TOF, Triple-quadropole mass spectrometry, Biomarkers, Neuroprognostication, Prognosis

## Abstract

**Background:**

Serum biomarkers may play a role in prognostication after cardiac arrest. This study was designed to assess the feasibility of using two-dimensional gel electrophoresis (2D-GE) coupled with mass spectrometry (MS) as a proteomic strategy to identify novel biomarkers that may predict neurological recovery.

**Methods:**

Adult comatose survivors of ventricular fibrillation or pulseless ventricular tachycardia were considered eligible. Blood was collected and serum separated within 6 h of hospital admission and then at 24 h afterwards. Neurological outcome was assessed at 3 months with the Cerebral Performance Category (CPC) score. Serum was assessed with 2D-GE with and without prior depletion of high abundance proteins. Protein differences between patients with good (CPC 1,2) vs. poor (CPC 3–5) neurological recovery were subsequently identified with MS.

**Results:**

From August 2010 to June 2014, 11 patients meeting eligibility criteria were recruited, from which serum was available from 9 (5 with good neurological outcome). On non-depleted serum, only high abundance acute phase proteins such as haptoglobin, cell-free hemoglobin, albumin, and amyloid were detected in both patients with good and poor neurological recovery. Following depletion of high abundance proteins, proteins identified by MS in both patient populations were the acute phase reactants c-reactive protein and retinol binding protein-4. Proteins uniquely identified in the serum of patients with poor neurological recovery included 14-3-3 (epsilon and zeta isoforms) and muskelin.

**Conclusions:**

Two-D-GE coupled with MS is a feasible strategy to facilitate the identification of novel predictive biomarkers. The presence of muskelin and 14-3-3 in the serum of patients with poor neurological prognosis warrants further investigation.

## Background

One of the most difficult challenges for physicians caring for patients that are resuscitated after cardiac arrest is providing accurate prognostic information regarding neurologic recovery. A combination of clinical, electrophysiological, and biochemical findings can help predict catastrophic outcomes (e.g., death or persistent vegetative state [1]). For example, bilateral absent pupillary light responses and corneal reflexes have false positive rates of 0.2 and 0.4 %, respectively, for predicting poor neurological recovery. However, it is common for survivors of cardiac arrest to remain comatose, but with present brainstem reflexes. For this cohort of patients, prognosis is particularly difficult, as neurological recovery can lie anywhere on the spectrum from normal to persistent vegetative state. Elevated serum levels of neuron-specific enolase have been found by some researchers to be a reliable indicator of poor neurological recovery [2], but not others [3, 4]. Therefore, the identification of novel serological biomarkers may facilitate accurate prognosis for these patients.

The use of proteomics in the intensive care unit to identify predictive biomarkers is gaining popularity in a number of clinical situations, particularly sepsis (reviewed in [5]). For example, two-dimensional gel electrophoresis (2D-GE) coupled with matrix-assisted laser desorption/ionization time of flight mass spectrometry (MALDI-TOF MS) identified protein biomarkers from serum samples that distinguished between patients who survived septic shock versus those who did not [6] and septic patients that required renal replacement therapy [7]. Our study was designed to assess the utility of 2D-GE coupled with MS as a feasible proteomic strategy to identify biomarkers that correlated with neurologic recovery following cardiac arrest. Our overall hypothesis was that this approach might identify novel protein biomarkers that distinguish between patients with good vs. poor neurological outcome after cardiac arrest.

## Methods

This prospective cohort feasibility study was conducted on patients admitted to the 33-bed intensive care unit at a tertiary care academic/teaching hospital. Our local Health Sciences Research Ethics Board approved the study protocol.

### Inclusion and exclusion criteria

Following informed consent from the substitute decision maker, we prospectively enrolled 11 consecutive patients who met the inclusion and exclusion criteria, which were similar to previous studies [8, 9]. Briefly, eligible patients were 18 years and older whom spontaneous circulation was restored <60 min following witnessed cardiac arrest due to ventricular fibrillation (VF) or pulseless ventricular tachycardia (VT). We also included patients who had shocks delivered by an automatic external defibrillator, as presumably, these patients had VT or VF. We chose to focus on this cohort of cardiac arrest patients, as the underlying etiology tends to be myocardial ischemia. In contrast, patients who have asystole or pulseless electrical activity may be more heterogeneous in nature. For example, we did not wish to include patients who had a hypoxemic cardiac arrest due to pneumonia and sepsis, as the underlying sepsis syndrome would likely alter their proteomic profile. Patients needed to be enrolled within 6 h of the cardiac arrest. Exclusion criteria included pregnancy, hypothermia on admission (tympanic temperature <30 °C), response to verbal commands following restoration of spontaneous circulation (i.e., the patient was not comatose), persistent hypotension despite fluids/pressors (mean arterial pressure (MAP) <60 mmHg) or hypoxia (O_2_ saturation <85 %) for more than 30 min after return of spontaneous circulation, a terminal illness that preceded the cardiac arrest, and poor baseline functional status (defined as modified Cerebral Performance Category (CPC) ≥3) prior to the cardiac arrest.

### Treatment

Eligible patients were treated with targeted temperature management using surface cooling as soon as possible. This included ice packs and a commercial cooling blanket. Target core temperature (32–34 °C) was maintained for 24 h, followed by passive rewarming. The hemodynamic parameters of each patient were managed at the discretion of the treating intensivist. Coronary revascularization procedures were also performed at the discretion of the treating attending intensivist and consultant interventional cardiologist.

### Clinical data collection

After an informed consent was obtained from the substitute decision maker, each enrolled patient was assigned a case response form. Basic demographic and health history was collected. During the patients’ ICU stay, we recorded clinical data, outcomes, and physiological parameters, including heart rate, mean arterial pressure, and temperature (measured rectally while the patient was undergoing therapeutic hypothermia, orally if patient was awake). To determine the patients’ 3-month outcome, the CPC was used, in line with other investigations of neurologic recovery after cardiac arrest [9]. The CPC is a 5-point scale, which ranges from 1 = none, or mild cerebral disability, 2 = moderate cerebral disability, 3 = severe cerebral disability, 4 = comatose, and 5 = dead. The CPC was dichotomized into good (CPC 1–2) or poor (CPC 3–5) outcomes as done previously [8, 9]. The CPC for each patient was determined either by telephone interview or chart review by the one of the study investigators (J.G.B.).

### Sample collection and proteomic separation with 2D-GE

Whole blood was collected at the time of admission to the ICU (within 6 h of cardiac arrest) and at 24 h. It was immediately transported to the core laboratory for centrifugation and isolation of serum using standard protocols. Serum was stored at −80 °C until proteomic assessment. To create a unique protein fingerprint, proteins were separated using 2D-GE as previously described [10, 11]. Briefly, equal quantities of serum proteins were loaded onto a pH (3–11) gradient strip overnight and then ran in an isoelectric focusing machine to separate the serum proteins as per their isoelectric focusing point. The immobilized pH gradient (IPG) strips containing protein were separated on a 12 % acrylamide gel at 4 **°**C overnight, washed, and then silver stained. A second analysis was performed on the serum after high abundance proteins were depleted using a commercially available high-performance liquid chromatography-based column according to the manufacturer’s instructions (Multiple Affinity Removal Column, Human 14; Agilent Technologies, Canada). This column depletes serum of the following high abundance proteins: albumin, IgG, antitrypsin, IgA, transferrin, haptoglobin, fibrinogen, alpha2-macroglobulin, alpha1-acid glycoprotein, IgM, apolipoprotein AI, apolipoprotein AII, complement C3, and transthyretin.

### Mass spectrometry

For non-depleted serum samples, MALDI-TOF MS was used to identify proteins of interest from the 2D gels as previously described [10]. To increase the ability to detect smaller quantities of protein in the depleted samples, triple quadrupole/linear ion trap MS was performed using the QTRAP 5500 System (Sciex, Canada) according to standard protocols. The data files were submitted to Mascot for the protein library search.

### Statistical analysis

To determine if there were significant differences between hemodynamic parameters between patients with good vs. poor neurological outcome, *t* tests were used. Results were considered significant if *p* < 0.05. Due to the feasibility nature of this project, no correction was performed for multiple comparisons, as all results are considered exploratory.

## Results

### Patients

From August 2010 to June 2014, a convenience sample of 11 patients were recruited into the study (Table 1). Of the 11 patients recruited, all but 2 were male. The median age was 61 years (range 27–76). Five of the patients had no known cardiac history. For patient 5, the presenting rhythm was initially interpreted as ventricular fibrillation, and the patient was recruited into the study. On further review of the cardiac arrest, the initial rhythm was not ventricular fibrillation, but a non-perfusing bradyarrhythmia that subsequently degenerated into ventricular fibrillation. The serum was collected, but subsequently discarded when it was identified that the patient did not specifically meet the inclusion criteria. Patient 9 was eligible for enrollment into the study, but due to a system error, his blood was not sent to the laboratory to have the serum separated. The remaining patients had serum collected and available for proteomic analysis. For the five patients with poor neurological outcomes, charts were reviewed to assess their best possible CPC score, in case they recovered neurologically but died of a non-neurological cause by 3 months. None of the five patients in the poor neurological recovery group regained consciousness at any time.

**Table 1:** Patient demographics and clinical characteristics

Patient #	Age/gender	Comorbidities	Rhythm	CPC
Good neurological outcome				
1	61 M	Obesity/OA	VF	1
2	76 F	Obesity, HTN, DM, AS, AF	PEA*	1
3	62 M	COPD, CKD, CAD, DM, CHF	VT	1
7	46 M	Healthy	VF	1
9	37 F	Endometriosis, nephrolithiasis, epilepsy	VT	1
10	57 M	Healthy	VF	1
Poor neurological outcome				
4	68 M	HTN, DM, smoker	VF	4
5	70 M	Healthy	VF	5
6	47 M	Healthy	VF	5
8	67 M	HTN, AF	VF	5
11	27 M	Healthy	VF/VT	5

The demographic information, initial rhythm, and 3-month neurological outcome are shown for the 11 patients recruited into the study

*CPC* cerebral performance category, *VF* ventricular fibrillation, *OA* osteoarthritis, *HTN* hypertension, *DM* diabetes mellitus, *AS* aortic stenosis, *AF* atrial fibrillation, *COPD* chronic obstructive pulmonary disease, *CKD* chronic kidney disease, *CAD* coronary artery disease, *CHF* congestive heart failure, *PEA* pulseless electrical activity, *VT* ventricular tachycardia.

* Patient was initially classified as having VF, but reclassified later as having a non-perfusing bradycardia (see text for details)

### Hemodynamics and thermal monitoring

**Fig. 1:**
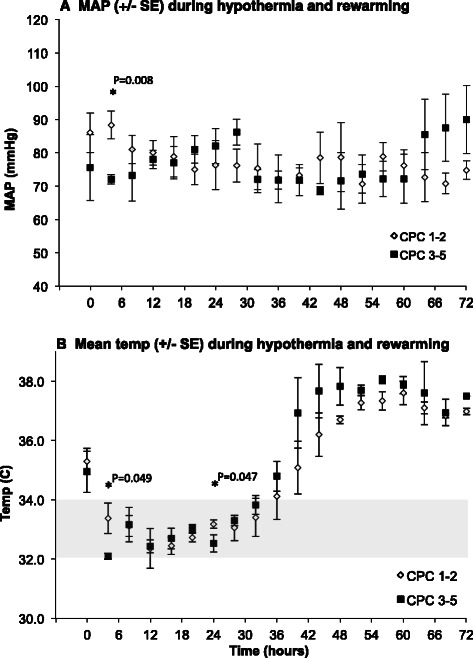
The mean value (+/− SEM) (**a**) of the mean arterial pressure (MAP) and body temperature (**b**) were recorded from the chart every 4 hours after admission to the intensive care unit. Patients with good neurological recovery (CPC 1–2) are represented by the *white diamonds*, whereas patients with poor neurological recovery (CPC 3–5) are represented by the *black squares*

The mean arterial pressure and heart rate for each patient was recorded from the chart at 4-h intervals. The mean MAP was above 65 mmHg for both groups of patients during the first 72 h of recording (Fig. 1a). Shortly after admission to the ICU, patients who ultimately attained good neurological recovery had a higher mean MAP than patients who had a poor neurological recovery. There was no significant difference in MAP between these two patient groups at any other time period. There was no difference in mean heart rate between patients with good vs. poor neurological recovery (data not shown). All patients reached their targeted temperature within 6 h of being admitted to the intensive care unit (<6 h after arrest). There was a trend for patients who ultimately had poor neurologic outcomes to cool more quickly and overshoot their temperature upon rewarming, consistent with prior reports of impaired thermoregulation among patients with poor neurologic prognosis (Fig. 1b, see [12]).

### Proteomics

**Fig. 2:**
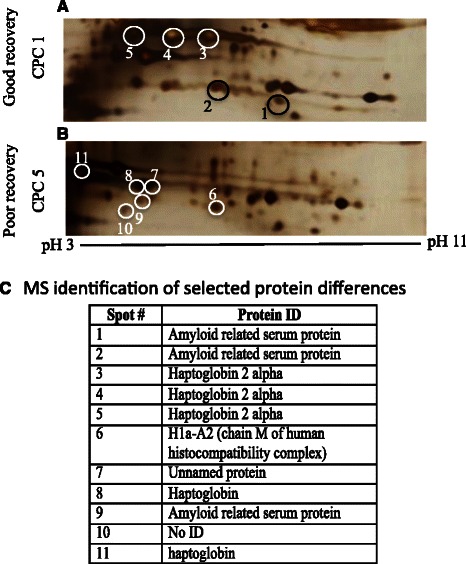
Non-depleted serum proteome of patients within 6 h of cardiac arrest demonstrates acute phase high abundance proteins. The serum proteome (pH 3–11) of a representative patient with good neurological recovery (CPC 1, **a**) after cardiac arrest has both unique and overlapping protein spots when compared to a patient with poor neurological recovery (CPC 5, **b**). **c** Eleven selected proteins that were visually different between these two patients were subsequently identified as high abundance proteins, such as amyloid-related serum protein and haptoglobin. The same protein identified at several different locations likely reflects post-translational modifications

As shown in Fig. 2, the serum proteomes of a patient with good neurological recovery (Fig. 2a) and a patient with poor neurological recovery (Fig. 2b) have both unique and overlapping characteristics. Selected protein spots were assessed with MALDI-TOF MS and were identified as various isoforms of amyloid and haptoglobin, as well as the H1a-A2 protein (Fig. 2c). Haptoglobin is also considered an acute phase reactant and is increased in patients after cardiac arrest [13]. In addition to these proteins, a common protein identified in the serum at 24 h of both patients with good and poor neurological recovery was cell-free hemoglobin (data not shown).

**Fig. 3:**
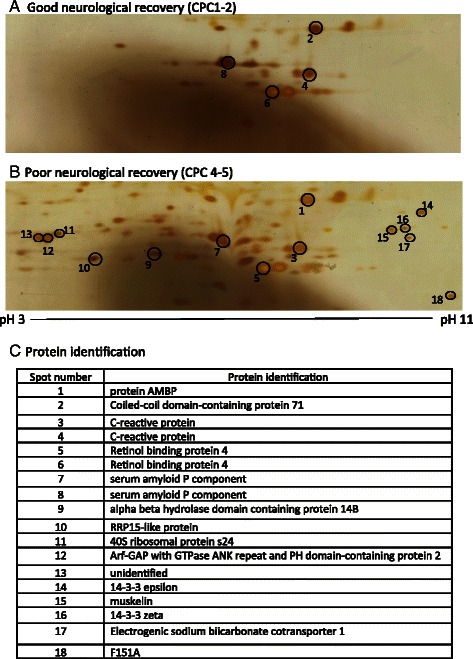
Depleted serum proteomes may demonstrate novel biomarkers of poor neurological recovery after cardiac arrest. Representative pooled serum proteomes taken within 6 h of cardiac arrest from patients with a good neurological recovery (CPC 1–2, **a**), have many fewer protein spots when compared to the pooled serum proteome from patients with poor neurological recovery (CPC 3–5, **b**). **c** Triple-quadrupole/linear ion trap MS identified 17 of 18 of these proteins

**Fig. 4:**
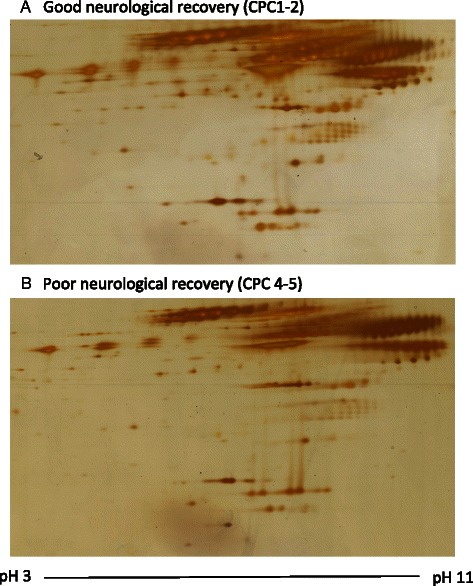
Pooled serum proteomes 24 h after cardiac arrest. Little differences were observed in the proteomic phenotype of the pooled serum from patients with good neurological recovery (CPC 1–2, **a**) compared to patients with poor neurological recovery (CPC 3–5, **b**). Therefore, no protein spots were isolated for MS

Since replicate gels (*n* = 3) all showed a similar pattern of high abundance, acute phase proteins, we elected to deplete the serum with a commercially available high performance liquid chromatography protein column. This process eliminates up to 90 % of the 12 most abundant serum proteins, including albumin, immunoglobulin, haptoglobin, etc. The resulting individual proteomes showed variable proteomic profiles at baseline and 24 h after cardiac arrest, in both patients with good and poor neurological outcome (not shown). MS was challenging on these samples because the volume of protein was so low, only a few scattered proteins could be identified. Two of these proteins may be markers of endothelial dysfunction, such as von Willebrand Factor and endothelin converting enzyme 1 (not shown). Therefore, we pooled the individual depleted serum samples into two samples, good vs. poor neurological recovery, and re-ran the protein separation with 2D gels. The baseline serum proteome (collected within 12 h of arrest) was markedly different between patients with good (Fig. 3a) and poor (Fig. 3b) neurological outcomes. Proteins commonly expressed in both groups were C-reactive protein, retinol binding protein-4, and serum amyloid P component (Fig. 3c). A number of proteins uniquely expressed by patients with poor neurological recovery were also identified with MS, including 14-3-3 (epsilon and zeta isoforms), Arf-GAP with GTPase ANK repeat and PH domain containing protein-2, and muskelin. At 24 h after cardiac arrest, very few differences were noted in the pooled/depleted proteomes, so MS was not performed (Fig. 4a, b).

## Discussion

This prospective cohort feasibility study recruited 11 consecutive patients following successful resuscitation from cardiac arrest to determine the feasibility of performing a proteomic assessment of patient sera. In the past, we have used a 2D-GE coupled with MALDI-TOF MS proteomic approach to identify novel biomarkers that distinguish between different types of glia in the nervous system [10, 11]. However, this is the first study to demonstrate that this is a feasible proteomic strategy that may be used to identify novel serum biomarkers that correlate with neurologic recovery after cardiac arrest.

The principal advantage to using this specific proteomic approach to search for predictive biomarkers is that it is not necessary to define a priori which biomarkers are being examined. For instance, most studies on prognostic biomarkers after cardiac arrest have focused on proteins released into the bloodstream upon damage to the nervous system, such as neuron-specific enolase (NSE) and S100. More recently, other putative biomarkers have been studied, such as B-type natriuretic peptide, s-selectin, cell-free DNA, and tau [3, 14–18]. Although these markers have shown promise in predicting poor outcomes in patients treated with therapeutic hypothermia, their sensitivity and specificity remain suboptimal. The unifying theme of these prior studies is that the protein was known ahead of time. Using serum proteomics as a “discovery-based approach” to biomarker research allows for the identification of proteins not previously considered important or relevant to the clinical situation. For example, we identified several putative biomarkers of poor neurological recovery, including Arf-GAP with GTPase ANK repeat and PH domain-containing protein 2, 14-3-3 proteins (episilon and zeta isoforms), and muskelin. Muskelin is of particular interest, as it is highly expressed in areas of the brain that are particularly susceptible to hypoxic-ischemic injury, namely the cerebellum and hippocampus [19], and appears to facilitate trafficking of GABA-A receptors [20].

Another advantage of the proteomic approach to biomarker discovery is the number of usable differences that are discovered. Unlike RNA- or DNA-based microarray technology that has the capacity to identify thousands of gene differences between samples, 2D-GE/MS generates a manageable number of protein differences. For example, the proteomic strategy used in our study has previously identified only four protein biomarkers from serum samples that distinguished between patients who survived septic shock versus those who did not; specifically, complement factor B, haptoglobin, and clusterin were decreased in non-survivors, whereas α-1-B-glycoprotein was elevated in survivors [6]. More recently, Gong and colleagues [7] demonstrated effective 2D-GE proteomic assessment of serum pooled from critically ill patients undergoing continuous renal replacement therapy for renal failure related to sepsis. Sera were collected before and at 24, 48, and 72 h after initiation of renal replacement therapy. The serum proteomes were compared to similar sepsis patients who did not require dialysis. Thirty-four spots (proteins) were identified by 2D-GE that changed significantly with treatment, of which MS successfully identified 10. Many of these identified proteins were associated with inflammation and coagulation, which might not have been considered in the context of sepsis and renal replacement. Although MS-based technology may not have the resolution to identify proteins in very small quantities or metabolites, such as high performance liquid chromatography (e.g., [21]), we anticipate that this technology will identify proteins that can be easily quantified with other platforms, such as enzyme-linked immunosorbent assays (ELISA).

One of the main limitations of this study is its small sample size. We recruited 11 patients, 9 of whom had serum available for proteomic analysis. The low recruitment rates are likely related to the very tight window for inclusion (within 6 h of cardiac arrest) and inability to enroll patients 24 h/day. Given that the main differences were identified at 6, but not 24 h, we would be reluctant to increase the period of time from arrest to first blood sample collection. While we are unable to draw any definitive conclusions about the proteins that have been identified, we have attained our primary outcome in determining the feasibility of identifying proteins from serum of critically ill patients after cardiac arrest. The positive results of this feasibility study have provided the justification for pursuing a larger proteomic investigation with a larger sample size. Should proteomic differences be identified with our qualitative MS protocol, the next step would involve confirmation and quantification, perhaps with ELISA-based techniques. Another limitation of this study is the gender difference between groups. In the patients with good neurological recovery, 4/6 were male. This is in comparison to the patients in the poor recovery group that were all male. Due to the small size of our study, we are unable to account for any possible gender differences on the proteomic profile. Similarly, our small sample size is the likely explanation for the fact that most patients had either a CPC of 1 (no neurological deficits) or 5 (dead) at 3 months. Furthermore, the treatment of patients, including their targeted temperature management, hemodynamic support, and end-of-life care was not protocolized. Future studies attempting to identify novel prognostic biomarkers with proteomic approaches would benefit from protocolized therapy for these patients.

## Conclusions

Providing accurate prognostic information to families of patients who have a cardiac arrest remains challenging. Clinicians may use a combination of clinical, radiological, and electrophysiological data to determine the neurologic prognosis for these patients. Biomarkers are less reliable, especially for patients treated with therapeutic hypothermia after cardiac arrest. However, this study demonstrates that 2D-GE coupled with MS is a feasible proteomic strategy to search for novel biomarkers that may correlate with prognosis after cardiac arrest and may be a strategy to identify key proteins that can contribute to the information a clinician requires to make an accurate neurologic prognosis after cardiac arrest.

## Abbreviations

2D-GE: two-dimensional gel electrophoresis
CPC: cerebral performance category
MALDI-TOF: matrix-assisted laser desorption/ionization time of flight
MAP: mean arterial pressure
MS: mass spectrometry
VF: ventricular fibrillation
VT: ventricular tachycardia
